# Social support and antenatal depression in extended and nuclear family environments in Turkey: a cross-sectional survey

**DOI:** 10.1186/1471-244X-11-48

**Published:** 2011-03-24

**Authors:** Vesile Senturk, Melanie Abas, Oguz Berksun, Robert Stewart

**Affiliations:** 1 Department of Psychiatry, Ankara University Medical School, Ankara, Turkey; 2 King's College London (Institute of Psychiatry), London, UK

## Abstract

**Background:**

Social support is strongly implicated in the aetiology of perinatal mental disorder: particularly the quality of the marital and family environment. Family structures are important under-researched potential modifiers. Turkey offers particular advantages for research in this area because of long-standing coexistence of Western and Middle Eastern family structures. We aimed to investigate associations between the quality of key relationships and depression in women in their third trimester of pregnancy, and the extent to which these associations were modified by family structure.

**Method:**

Women attending antenatal clinics in their third trimester were recruited from urban and rural settings in Ankara. A nuclear family structure was defined as a wife and husband living alone or with their children in the same household, whereas a traditional/extended family structure was defined if another adult was living with the married couple in the same household. Depression was ascertained using the Edinburgh Postnatal Depression Scale (EPDS) and social support was assessed by the Close Person Questionnaire with respect to the husband, mother and mother-in-law. Social support was compared between participants with/without case-level depression on the EPDS in linear regression models adjusted for relevant covariates, then stratified by nuclear/traditional family structure.

**Results:**

Of 772 women approached, 751 (97.3%) participated and 730 (94.6%) had sufficient data for this analysis. Prevalence of case-level depression was 33.1% and this was associated with lower social support from all three family members but not with traditional/nuclear family structure. The association between depression and lower emotional support from the husband was significantly stronger in traditional compared to nuclear family environments.

**Conclusions:**

Lower quality of relationships between key family members was strongly associated with third trimester depression. Family structure modified the association but, contrary to expectations, spousal emotional support was a stronger correlate of antenatal depression in traditional rather than nuclear family settings. Previous psychiatric history was not formally ascertained and the temporal relationship between mood state and social support needs to be clarified.

## Background

Common mental disorders have a high prevalence in women, particularly at childbearing age [1]. It is increasingly recognised that many, if not the majority of cases of perinatal depression begin in the antenatal period and persist after childbirth [2]. Perinatal depression is a major health issue for many women from diverse cultures, although most often investigated in the postnatal rather than antenatal period [3,4].

A meta-analysis of 59 studies reported a postnatal depression prevalence of 13% [5], although this varied widely between studies and was found to depend substantially on the instrument and criteria used. British and Swedish studies have reported high maintenance rates (33-37%) and relatively low incidence rates (5-7%) of depression from the antenatal to postnatal periods [6,7]. Antenatal depression is therefore an important risk factor for postnatal depression and perinatal depression, whether antenatal or postnatal, represents an important public health problem [8,9]. However, research into antenatal depression has been very limited. It has been estimated that the prevalence of antenatal depression may be as high as 20% [10,11], although a systematic review by Bennett and colleagues estimated a prevalence of 12% in the third trimester [12]. Predisposing factors include a previous history of depression or mental disorder [10], but social support is also strongly implicated in the aetiology of perinatal mental disorder with the quality of the marital and family environment particularly pertinent [13-15]. The majority of research in this area has been carried out in Western settings with relatively homogeneous family structures and limited generalisability to the rest of the world.

Throughout the 20th century, Turkey has experienced substantial demographic, sociocultural and economic transformations. These changes have been said to be linked with adverse consequences such as poverty, unemployment, limited social services, and an imbalance in income distribution [16,17]. Taken together, these changes can be supposed to have an important impact on maternal health in the antenatal period that could be mediated through loss of traditional support networks. However, little is known about antenatal depression in Turkey, although postnatal depression has been found to be common [18], and Turkish research has generally confirmed risk factors suggested from Western populations such as low income and socio-economic status, previous mental disorder, recent life events and perceived poor child health[19]. Level of social support has also been implicated in several studies: not only that from the husband [19,20] but also from the husband's family and the wife's parents [20]. Although traditional family relationships in Turkey are believed to be strong, Inandi et al [21] observed that almost 40% of women complained of insufficient family support during pregnancy. Golbası et al also found a moderate negative correlation between depression and perceived social support as well as positive correlations with maternal age, gravidity and number of living children [22].

A particularly important potential consequence of global population expansion and trans-national and rural-urban migration has been the disruption of traditional family-based support structures common in Middle Eastern, as well as other societies. However, Turkey is almost unique as a nation in the length of time over which, and in the geographic proximity within which, both modern Western ('nuclear') and traditional Middle Eastern ('extended') family structures have co-existed. Comparisons between different family structures are important for women's mental health because of the rapid 'Westernisation' of families occurring in many settings around the world and Turkish culture offers a particularly informative setting to investigate the longer term impact of these transitions.

In the context of an ongoing prospective study of social support and perinatal depression carried out in Ankara, we analysed baseline data to investigate associations between the quality of family relationships and depression in women in their third trimester of pregnancy. Taking Turkish culture into account, the research was specifically focused on the quality of three key relationships for a woman expecting children: i.e. with her spouse, with her mother and with her mother in- law. We further sought to investigate the extent to which these associations were modified by family structure. The following *a priori *hypotheses were formulated for the baseline analyses of this study: 1) ante-natal depression will be associated independently with reduced reported quality of relationship between the woman, her husband, her mother and her mother-in-law; 2) these associations will be evident for all three derived subscales of the Close Persons Questionnaire (emotional support, practical support and negative aspects of the relationship); 3) these associations will differ between traditional and nuclear family settings: in particular the association with lower quality of the spousal relationship will be stronger in nuclear family settings (anticipating that traditional structures, with the presence of other potentially supportive family members, may provide a buffering role for this association).

## Methods

### Setting

The study was carried out in and around Ankara, the capital of Turkey, an appropriate setting because of the considerable heterogeneity of the population in terms of traditional Middle Eastern or 'modern' Western lifestyles and social environments. 'Ankara' here includes both central urban and semi-rural locations. In common with other Turkish cities, it has experienced rapid expansion and immigration. Many young women living in urban districts have migrated as students or working adults and live a long distance away from their parents. On the other hand, in the surrounding more rural districts, women will be more likely to be cohabiting with their family with traditional ties and expectations.

### Participants

Samples were drawn from urban and rural antenatal clinics in and around Ankara. These clinics were purposively selected to maximise population heterogeneity as it was not feasible to carry out a formal random sampling process. Attempts were made to interview all attenders for routine third trimester antenatal examinations within the study period from December 2007 to August 2008. Usual clinic attendance is at around 32 weeks. Attendance for routine perinatal services are very high in Ankara: for example, attendance for measles and BCG vaccinations 93% and 92% respectively [23]. After approach, agreement and written informed consent, a brief interview was administered by a research team of trained interviewers (graduate-level research workers and trainee psychologists) at the time of clinic attendance.

Participants did not receive a payment and, as part of the consent process, were made aware that they were free to refuse the interview or any component of this without providing a reason and without any effect on their healthcare. Refusal reasons were mainly lack of time or non-permission of the husband. Women with depressive symptoms were not formally re-evaluated clinically. However, women with moderate or severe depressive symptoms and wishing treatment were referred to their GP or to a psychiatrist.

### Measurements

Depressive symptoms were ascertained using the Edinburgh Postnatal Depression Scale (EPDS), the most widely used screening instrument for perinatal depression in both international and Turkish research. It focuses on cognitive symptoms of depression and excludes somatic items which may generate false positive cases in pregnancy and *post partum *[24]. It is a 10-item self-report measure with 0-3 scores for each item, giving a potential scale score range of 0-30, and has been validated in many settings including in Europe [25], Africa [26], and America [27]. The reliability and validity study of the scale in Turkish was established [28] using the SCID as a gold standard, finding sensitivity and specificity of 0.76 and 0.71 respectively. In another validation study in Turkey [29] sensitivity was found to be 0.84 and specificity 0.88. In both studies, the optimum cut-off point for caseness was calculated to be ≥13. The EPDS has also been validated as a screening tool for antenatal depression [30]. The ≥13 cut-off was applied in our study to define case level depressive symptoms (hereafter referred to as 'depression').

Quality of individual relationships was measured using the Close Persons Questionnaire (CPQ) [31]. This is a widely applied instrument which focuses on three aspects of the quality of individual relationships - i) emotional support; ii) practical support; iii) negative aspects of the relationship (i.e. aspects of the relationship felt by the participant to make life more difficult for them). In a departure from the standard application of this instrument (where participants are asked to choose their most salient relationships to be rated), the index relationships were imposed so that questions were asked specifically and solely about the spouse, mother, and mother-in-law. Data were coded as missing on these sections if this information could not be obtained (e.g. if the mother or mother-in-law was deceased).

Other covariates in this analysis were as follows: 1) age, 2) number of living children, 3) education (self-reported, four groups), 4) family income (four groups), 5) self reported general physical health (five groups), 6) presence of self-reported life stressors/events (debt, hunger from lack of food, recent separation, problems with friends, recent illness/assault, violence to self, illness in a relative, death of a close family member, death of another relative, problems with a job, problems with money, problems with the justice system, any robbery), 7) self-reported past history of emotional problems, 8) family structure.

Family structure was defined as an effect modifier for analyses and was applied as a binary variable, categorising into nuclear or traditional/extended family structure. A nuclear family structure was defined as a wife and husband living alone or with their children in the same household, whereas a traditional/extended family structure was defined if another adult was living with the married couple in the same household. In Turkish society this would nearly always be the mother-in-law and/or father in-law of the woman since it is near-universal practice in traditional settings for women, following marriage, to live with their spouse's family (i.e. it was not anticipated that there would be any families where the woman and her spouse were living with her own parents).

### Statistical analysis

A target sample size of 750 women was calculated with the prospective study in mind, assuming a prevalence of 25% for case-level depression on the chosen scale at baseline, a maintenance rate of 30% through to the postnatal period, and a 0.5 standard deviation group difference in mean score for a given quality of relationship measure between maintained and non-maintained groups at 80% power (alpha 0.05, 2-sided test). At the same level of power, this sample size was calculated as allowing the detection of a 0.3 standard deviation group difference between participants with and without case-level depression at baseline, assuming a more conservative 13% prevalence.

The sample was initially described with respect to the covariates and associations between these and depression (EPDS caseness) was expressed through odds ratios and assessed using chi-squared tests. Although caseness on the EPDS was the primary 'outcome', in order to make use of the continuously distributed data on social support, the CPQ subscales were treated as dependent variables (i.e. testing the differences in social support scale means between participants with or without case-level depression) using t-tests initially to investigate significance. We then opted to use linear regression models to adjust for covariates. The sample size was felt to be sufficient to justify this approach of linear modelling, despite non-normal CPQ subscale distributions. Separate regression models were used for each CPQ subscale as a dependent variable with EPDS caseness entered and assessed as a binary independent variable on each iteration. Covariates were entered sequentially in the following groups: i) Model 1 adjusting for age only; ii) Model 2 adjusting for age, parity, education and family income; iii) Model 3 adjusting for age, parity, education, family income, physical health and number or life stressors/events; iv) Model 4 adjusting for age, parity, education, family income, physical health, number of life stressors/events and self-reported previous emotional problems. As a secondary analysis, the fully adjusted model (Model 4) was re-run with EPDS score as a continuously distributed independent variable to check for linearity of associations. Stratified analyses were used to investigate effect modification by family structure with interaction terms re-tested in linear regression models. In a more exploratory analysis, effect modification by the presence or not of previous childbirth was investigated in a similar way through separate models.

## Results

Of the 772 women approached in their third trimester, 751 (97.3%) participated in the study. The reasons for non-participation were: refusal (n = 18) and insufficient literacy (n = 3). Thirty-one incomplete questionnaires had to be further excluded. Therefore, 730 (94.6%) were included with sufficient data for this analysis. The numbers of participants with complete data on the emotional support, practical support and negative aspects measures were 665, 670 655 for the mother respectively and 635, 649 and 633 for the mother in-law respectively. All 730 had spousal data.

### Sample characteristics

Distributions of covariates are summarised in the first column of Table 1. The mean age was 25.9 years (SD 5.3, range 18-44), and the mean duration of education was 8.4 years (SD 4.5, range 1-34). Almost all participants were living with their husband and close to a third (29%) were living in traditional family environments. Over half (53%) had no children. The majority (80%) described their physical health as at least good, although emotional problems in the past were reported by around half (49%) of the sample and the prevalence of reported violence in the last 12 months was 6%. Around a third (33%) had depression according to the EPDS≥13 cut-off point.

**Table 1:** Unadjusted associations between participant characteristics and prevalence of case-level depressive symptoms

	n	Depression prevalence (%)	ORs (CIs)	χ^2 ^(df), p-value
Age				0.03 (1) p = 0.86
18-22	199	33.7	Reference	
23-25	166	37.3	1.18 (0.76-1.81)	
26-44	172	25.0	0.66 (0.42-1.03)	
30-44	168	36.9	1.15 (0.75-1.77)	
Number of children				4.90 (1) p = 0.03
0	379	31.4	Reference	
1	230	29.6	0.92 (0.64 - 1.31)	
≥2	111	45.9	1.86 (1.21 - 2.86)	
Education level (year)				0.14 (1) p = 0.71
≤5	229	32.3	Reference	
6-8	143	32.9	1.03 (0.66 - 1.60)	
9-11	240	34.2	1.09 (0.74 - 1.60)	
11≥	82	26.8	0.77 (0.44 - 1.35)	
Family income (TRY)				3.09 (1) p = 0.08
≤630	169	36.7	Reference	
631-900	172	36.0	0.97 (0.63 - 1.51)	
901-1400	243	29.6	0.73 (0.48 - 1.10)	
1401- 23000	93	29.0	0.71 (0.41 - 1.22)	
Physical health				9.33 (1) p < 0.001
Very good	129	30.2	Reference	
Good	446	29.6	0.97 (0.63 - 1.49)	
Average/bad/very bad	142	47.2	2.06 (1.25 - 3.40)	
Life events/stressors				43.7 (1) p < 0.001
0	406	23.6	Reference	
1	174	31.0	1.46 (0.96 - 2.20)	
2	90	43.0	2.45 (1.49 - 4.03)	
3	69	55.0	3.96 (2.02 - 7.77)	
4+	27	70.4	7.70 (3.24 - 18.29)	
Past emotional problems				71.0 (1) p < 0.001
No	358	18.7	Reference	
Yes	340	48.8	4.14 (2.95 - 5.82)	
Family structure				0.08 (1) p = 0.77
Nuclear	471	32.7	Reference	
Traditional	249	33.7	1.05 (0.76-1.45)	

### Associations between covariates and depression

Unadjusted associations with depression are summarized in the remainder of Table 1. Depression was associated with higher numbers of previous children, worse general health, previous/current life events/stressors, and self-reported past history of emotional problems. There were no significant associations with age or education level. Depression was associated with lower family income, although only at borderline significance levels.

### Associations between depression and social support

Differences in social support measures between participants with or without depression are summarised in Table 2. In summary, women with case level depression reported worse social support (lower emotional and practical support, higher negative aspects of relationships) on all nine variables, apart from a lack of association with practical support from the mother.

**Table 2:** Unadjusted associations between social support and depressive symptoms

Nature of support	Mean (SD) social support		Difference (cases vs. non-cases)	
	Non-cases n = 482	Cases n = 238	Beta coefficient (95% CI)	p-value
*From husband*				
Emotional	19.4 (4.6)	15.4 (6.1)	-4.0 (-4.8, -3.2)	< 0.001
Practical	7.0 (2.0)	5.9 (2.4)	-1.1 (-1.4, -0.7)	< 0.001
Negative aspects	6.2 (2.2)	7.8 (2.2)	1.6 (1.2, 1.9)	< 0.001
*From mother*				
Emotional	17.1 (5.3)	15.3 (6.0)	-1.8 (-2.7, -0.9)	< 0.001
Practical	5.5 (2.8)	5.4 (3.1)	-0.1 (-0.6, 0.4)	0.62
Negative aspects	5.3 (2.1)	6.0 (2.2)	0.7 (0.3, 1.0)	< 0.001
*From mother in law*				
Emotional	11.3 (7.0)	6.9 (6.2)	-4.4 (-5.5, -3.2)	< 0.001
Practical	4.3 (3.1)	3.0 (3.0)	-1.3 (-1.8, -0.8)	< 0.001
Negative aspects	5.5 (2.4)	6.3 (2.9)	0.8 (0.3, 1.2)	< 0.001

Adjusted associations between depression and social support measures are displayed in Table 3. Adjustment for age had little impact on these, but there were modest reductions in the strengths of association following adjustment for number of children, duration of education, and family income. Further reduction was observed, particularly for the emotional support measures after adjustment for physical health and number of life events/stressors, with little or no subsequent change following adjustment for self-reported past emotional problems. In the final, fully adjusted model, depression remained significantly associated with all three measures of social support from the husband, with lower practical and emotional support from the mother-in-law and with higher negative aspects of the relationship with the mother. A secondary analysis, entering EPDS score as a continuous independent variable (rather than a binary case vs. non-case variable), gave essentially similar findings with significant negative correlations of EPDS score with emotional and practical support from the spouse and mother-in-law and emotional support from the mother. Significant positive associations were found with negative aspects of the relationship with all three relatives (data available on request).

**Table 3:** Adjusted associations between social support and depressive symptoms

Nature of support	Association with case-level depressive symptoms (B-value, 95% CI)				
	Unadjusted	Model 1	Model 2	Model 3	Model 4
*From husband*					
Emotional	-4.0 (-4.8, -3.2)*	-4.0 (-4.8, -3.2)*	-3.7 (-4.6, -2.9)*	-2.9 (-3.8, -2.0)*	-2.6 (-3.6, -1.7)*
Practical	-1.1 (-1.4, -0.7)*	-1.1 (-1.4, -0.7)*	-0.9 (-1.3, -0.6)*	-0.7 (-1.1, -0.3)*	-0.6 (-1.0, -0.2)*
Negative aspects	1.6 (1.2, 1.91)*	1.5 (1.2, 1.9)*	1.5 (1.1, 1.9)*	1.3 (0.9, 1.7)*	1.3 (0.8, 1.7)*
*From mother*					
Emotional	-1.8 (-2.7, -0.9)*	-1.8 (-2.7, -0.9)*	-1.6 (-2.6, -0.7)*	-1.0 (-2.0, 0.1)	-1.2 (-2.3, 0.2)
Practical	-0.1 (-0.4, 0.6)	-0.1 (-0.6, 0.4)	0.2 (-0.3, 0.6)	0.3 (-0.3, 0.8)	0.3 (-0.3, 0.8)
Negative aspects	0.7 (0.3, 1.0)*	0.6 (0.3, 1.0)*	0.7 (0.3, 1.1)*	0.7 (0.3, 1.1)*	0.7 (0.2, 1.1)*
*From mother in law*					
Emotional	-4.4 (-5.5, -3.3)*	-4.4 (-5.5, -3.3)*	-4.3 (-5.5, -3.2)*	-3.8 (-5.1, -2.6)*	-2.6 (-4.6, -1.9)*
Practical	-1.3 (-1.8, -0.8)*	-1.3 (-1.8, -0.8)*	-1.2 (-1.7, -0.7)*	-1.0 (-1.5, -0.5)*	-0.8 (-1.4, -0.3)*
Negative aspects	0.8 (0.3, 1.2)*	0.8 (0.3, 1.2)*	0.8 (0.4, 1.3)*	0.7 (0.2, 1.2)*	0.4 (-0.1, 1.0)

*p < 0.05

Model 1 Adjusted for age.

Model 2 Adjusted for 1 and number of children, duration of education, family income

Model 3 Adjusted for 2 and physical health, number of life stressors/events

Model 4 Adjusted for 3 and previous emotional problems

### Effect modification by family structure and previous childbirth

Stratified analyses investigating effect modification are summarised in Table 4. Overall, the associations between depression and social support were not modified substantially by family structure - in particular, there was no evidence for differences in the role of relationship with the mother-in-law, which appeared equally strong in both environments. Contrary to the *a priori *hypothesis, associations with spousal relationship were stronger in traditional compared to nuclear family settings: significantly so for lower emotional support as a correlate and of borderline significance for practical support. Also of borderline statistical significance was an apparent difference in maternal relationship as a correlate, depression being associated with lower practical support from the mother in a nuclear family setting but with higher practical support from the mother in a traditional family setting. Considering previous childbirth as an effect modifier, the association between depression and lower emotional support from the husband was stronger for women with no previous children; however, associations did not differ between groups with respect to other components of the spousal relationship or with quality of the other two relationships.

**Table 4:** Stratified analysis of associations between social support and depressive symptoms. B-coefficients with 95% confidence intervals are displayed

	Total	Family structure			Current family size		
		Nuclear n = 471	Traditional n = 249	p-value*	0 child n = 379	1+ children n = 341	p-value*
*From husband*							
Emotional	-4.0 (-4.8, -3.2)	-3.2 (-4.2, -2.2)	-5.4 (-6.8, -4.1)	< 0.01	-4.7 (-5.8, -3.6)	-3.2 (-4.3, -2.0)	0.05
Practical	-1.1 (-1.4, -0.7)	-0.8 (-1.3, -0.4)	-1.4 (-1.2, -0.9)	0.10	-1.1 (-1.5, -0.6)	-1.0 (-1.5, -0.5)	0.44
Negative aspects	1.6 (1.2, 1.9)	1.6 (1.2, 1.1)	1.4 (0.9, 2.0)	0.58	1.5 (1.0, 2.0)	1.6 (1.1, 2.2)	0.28
*From mother*							
Emotional	-1.8 (-2.7, -0.9)	-1.5 (-2.6, -0.4)	-2.3 (-3.9, -0.8)	0.37	-1.6 (-2.7, -0.5)	-1.9 (-3.3, -0.5)	0.24
Practical	-0.1 (-0.4, 0.6)	-0.4 (-1.0, 0.2)	0.5 (-0.3, 1.2)	0.07	0.6 (0.0, 1.1)	-0.7 (-1.4, 0.0)	0.10
Negative aspects	0.7 (0.3, 1.0)	0.7 (0.2, 1.1)	0.7 (0.1, 1.3)	0.95	0.6 (0.1, 1.1)	0.8 (0.2, 1.3)	0.18
*From mother in law*							
Emotional	-4.4 (-5.5, - 3.2)	-4.0 (-5.4, -2.6)	-5.1 (-6.9, -3.3)	0.34	-4.6 (-6.1, -3.0)	-4.0 (-5.5, -2.4)	0.42
Practical	-1.3 (-1.8, -0.8)	-1.3 (-1.9, -0.7)	-1.4 (-2.2, -0.7)	0.74	-1.1 (-1.8, -0.5)	-1.4 (-2.1, -0.7)	0.49
Negative aspects	0.8 (0.3, 1.19)	0.8 (0.2, 1.3)	0.7 (0.1, 1.4)	0.90	0.8 (0.3, 1.4)	0.7 (0.1, 1.4)	0.56

*Testing heterogeneity of regression coefficients between strata

## Discussion

In a sample of 730 women in their third trimester of pregnancy recruited in and around Ankara, we found that lower quality ratings for three key relationships - with the spouse, mother and mother-in-law - were associated with case level depression as defined from the Edinburgh Postnatal Depression Scale (EPDS). The association between lower quality spouse relationship and depression was stronger in women living in a traditional rather than nuclear family arrangement.

As discussed earlier, antenatal depression persists into the postnatal period in a large proportion of cases and many cases of postnatal depression begin in the antenatal period [6,7]. Antenatal depression therefore represents an important clinical and public health issue [8,9] because of the potential for early intervention. Although there have been reports of puerperal psychosis preceded by antenatal depression [32], postnatal depression as the much more common outcome is likely to be the most important focus for prevention. However, as discussed, there has been relatively little research into antenatal depression and its correlates. Findings from this study show similarities and dissimilarities compared to other studies in terms of potential risk factors for antenatal depression. Social support, life events, violence were associated with depression in one study whereas age, education level and income were not [33,34]. In another study, risk factors for depression during pregnancy included younger age and lower education [35,36]. Increased parity and lack of support, particularly poor support from the partner/husband have also been associated with depression in both developed and developing countries [34,37]. In particular, physical abuse by intimate partners before or during pregnancy has been found to be a particularly important potential risk factor for antenatal depression [35,36]. In this analysis, the focus was on social support and relationship quality specifically; however, unadjusted analyses revealed associations with increased parity, worse self-rated physical health, higher number of life events and self-reported past emotional problems. On the other hand, depression was not significantly associated with age, education, income or traditional/nuclear family structure. These findings suggest at least some level of heterogeneity between settings in correlates of antenatal depression, although methodological differences with respect to sampling and measurement cannot be excluded as an underlying reason for this.

Although it was not the primary objective of this analysis to investigate differences in the prevalence of antenatal depression between traditional and nuclear family settings, the observed lack of difference is potentially interesting. In the Turkish context, the two family models have co-existed for many decades and there is relatively little stigma attached to women living in either family model. In particular, we do not feel that women in nuclear settings have had to 'extract' themselves from traditional settings and thus we do not feel that the woman's personality or the attitudes of her family are likely to be a major factor. Largely, the family model in which a woman is living depends on issues such as the availability of work and accommodation. Further analysis would be required to clarify whether there was any negative confounding, obscuring a true difference in depression prevalence between settings. However, this was beyond the scope of this paper which sought to focus on associations between depression and social relationships and the role of family structure as an effect modifier rather than as an exposure itself.

To our knowledge, ours is the first study which has assessed the association between antenatal depression and support from the mother and mother in-law, although this has been investigated previously for postnatal depression in Turkey [36]. In our sample we found strong associations between depression and nearly all measures of social support from the three relatives in question. Those with the husband and mother-in-law were particularly strong, which is consistent with the importance of these figures in women's lives in this culture. The only exception was that negative aspects of the relationship with the mother were more strongly associated with depression than those with the mother-in-law. However, this might possibly reflect a long-standing poor parental relationship prior to marriage but with lasting effects on mental health.

As mentioned earlier, Turkey in general (and Ankara in particular) offers important advantages for research into the role of different family structures because of the longstanding co-existence of 'Western' and traditional 'Middle Eastern' cultures. The relationship between women and their mother and mother in-law is still important in Turkish culture, whether the woman is living in a nuclear or extended family setting. In Turkish traditional settings, a woman will typically move to live with her husband and his family in the same house when she gets married. In this setting, the expected role of a woman's own mother is to support this marriage by helping her daughter on practical issues (e.g. taking care of children) and emotional issues. The study was specifically set up to investigate these issues, funded through the Wellcome Trust's 'Health Consequences of Population Change' programme which sought to support research into the potential health impacts of rapidly changing societies. We investigated whether an extended family setting might modify potential effects of spousal and other key relationships on depression risk, specifically hypothesising that the presence of other family members would reduce the impact of a poor quality spousal relationship. Contrary to our hypothesis, effect modification in the opposite direction was found with stronger associations between spousal support and depression in traditional families, particularly with respect to lower emotional support as an exposure. This requires confirmation in other samples. However, it may reflect a higher visibility of marital difficulties in extended families and hence a stronger impact on depression. It might also reflect families taking the side of the husband and feelings of isolation of the woman in question. The stronger association in women without previous children might reflect a buffering effect of other children on the impact of marital strain or possibly higher feelings of empowerment in this group of women and/or the presence of children allowing greater access to friends and extra-familial support networks.

Also of interest was the observation that the association with social support from the mother-in-law was equally strong in nuclear and traditional families, emphasising the importance of this relationship in Turkish culture, and with implications for future clinical and public health interventions. The association with support from the mother was, as mentioned, weaker in most respects, and the observation of possibly opposite associations with daughters' depression between traditional and nuclear families might reflect differing roles of the mother in the two situations. Higher practical support from the mother in the context of an extended family structure (i.e. for women living with their husband's family) might represent a more severe breakdown of relationships in the household where women are residing. Support from family members has been found to be an important buffer against depression in women from other low and middle income settings [37]. Some research into perinatal mental disorder in Islamic nation settings has suggested both high prevalence of disorder and a potentially harmful role of disruptions to traditional family structures [38]. Although a high prevalence of antenatal depression was found in our sample, consistent with this, there was little evidence that traditional family structures conferred additional protection, either directly or through buffering effects of individual relationships. However, it should be borne in mind that these nuclear and traditional structures have co-existed in Turkey for a long time, potentially allowing individual and societal adjustment. Results cannot necessarily be generalised to nations or cultures undergoing more rapid changes and further research is required in these settings.

Strengths of this study include the particular features of the setting, as mentioned, the large and heterogeneous sample, the standardised assessment instruments which have been well-validated in a variety of international settings, and a comprehensive range of covariates. Random sampling of antenatal clinics was not feasible in this setting because of difficulties in enumeration of these. An approach was taken instead to maximise the heterogeneity of populations served which we believe constituted the next best approach to sampling. Response rates were relatively high and we believe that the findings should generalise to the source populations. The Edinburgh Postnatal Depression Scale used in this study is, as stated, widely used in international research. However, it should be borne in mind that it is a screening instrument, measuring number of depressive symptoms and does not seek to define specific depression syndromes. Furthermore, it is possible that other syndromes such as anxiety may influence caseness on this instrument. 'Depression' is therefore used as a shorthand term to describe case level symptomatology on this instrument, but it should be borne in mind that this is not synonymous with a clinical diagnosis and that clinical. Other principal limitations arise from the cross-sectional nature of this analysis. In particular, associations between lower social support and depression might reflect response bias in people with depression, or might reflect an effect of depression (the current episode and/or earlier episodes) on interpersonal relationships and actual levels of support, as well as the causal relationship of interest between low social support and risk of depression. In this respect, a key limitation is that there was little information on history of previous depressive episodes, whether known or unknown to clinical services, and further follow-up of this sample is currently underway which will seek to address these issues. Confounding factors were addressed as comprehensively as possible; however, residual confounding cannot be excluded. For example, personality was not measured although this is a factor that could have potentially influenced interpersonal relationships as well as risk of depressive episodes.

## Conclusions

In a large community sample of Turkish women in their third trimester of pregnancy, strong associations were found between depression and lower measures of social support from the husband, mother and mother-in-law. Taking advantage of the wide range of family structures of participants in this setting, we investigated the modifying role these might have on the associations of interest. In summary, and contrary to our hypothesis, we found that lower quality of the spousal relationship had stronger rather than weaker associations with depression in traditional, extended family settings. Lower quality of emotional support from the husband was also more strongly associated with depression in women with no previous children.

There has been considerable concern around changes in family structures over the last 100 years and their impact on mental health. Turkey, in common with other Middle Eastern countries has been particularly affected, although changes have been occurring over a relatively longer period. To our knowledge, ours is the first study to investigate the role of the family structure and social support within the family as aetiological factors for antenatal depression and we believe that our findings provide a template for further research both in Turkey and elsewhere.

## List of abbreviations

(EPDS): Edinburgh Postnatal Depression Scale; (CPQ): Close Persons Questionnaire; (SD): Standard deviation; (95% CI): 95% confidence intervals.

## Declaration of Competing interests

The authors declare that they have no competing interests.

## Authors' contributions

Funding for the study, as designed, was obtained by VS, RS and OB. The study from which data are reported was carried out by VS under the supervision of RS, MA and OB. Data were analysed by VS who prepared this report to which RS, MA and OB contributed critical comment. All authors read and approved the final manuscript.

## Pre-publication history

The pre-publication history for this paper can be accessed here:

http://www.biomedcentral.com/1471-244X/11/48/prepub
